# Oxidative Stress and Inflammation in B-Cell Lymphomas

**DOI:** 10.3390/antiox12040936

**Published:** 2023-04-15

**Authors:** Mário Sousa-Pimenta, Maria Manuela Estevinho, Miguel Sousa Dias, Ângelo Martins, Letícia M. Estevinho

**Affiliations:** 1 Department of Onco-Hematology, Portuguese Institute of Oncology of Porto (IPO-Porto), 4200-072 Porto, Portugal; msousapimenta@ipoporto.min-saude.pt (M.S.-P.);; 2 i3S—Instituto de Investigação e Inovação em Saúde, Universidade do Porto, 4200-135 Porto, Portugal; 3 Department of Gastroenterology, Vila Nova de Gaia/Espinho Hospital Center, 4434-502 Vila Nova de Gaia, Portugal; 4 Department of Biomedicine, Unit of Pharmacology and Therapeutics, Faculty of Medicine, University of Porto, 4200-319 Porto, Portugal; 5 Mountain Research Center (CIMO), Polytechnic Institute of Bragança, 5300-252 Bragança, Portugal; 6 Department of Biology and Biotechnology, Agricultural College of Bragança, Polytechnic Institute of Bragança, 5300-252 Bragança, Portugal

**Keywords:** inflammation, lymphoma, reactive oxygen species

## Abstract

Mature lymphoid neoplasms arise de novo or by the transformation of more indolent lymphomas in a process that relies on the stepwise accumulation of genomic and transcriptomic alterations. The microenvironment and neoplastic precursor cells are heavily influenced by pro-inflammatory signaling, regulated in part by oxidative stress and inflammation. Reactive oxygen species (ROSs) are by-products of cellular metabolism able to modulate cell signaling and fate. Moreover, they play a crucial role in the phagocyte system, which is responsible for antigen presentation and the selection of mature B and T cells under normal conditions. Imbalances in pro-oxidant and antioxidant signaling can lead to physiological dysfunction and disease development by disrupting metabolic processes and cell signaling. This narrative review aims to analyze the impact of reactive oxygen species on lymphomagenesis, specifically examining the regulation of microenvironmental players, as well as the response to therapy for B-cell-derived non-Hodgkin lymphomas. Further research is needed to investigate the involvement of ROS and inflammation in the development of lymphomas, which may unravel disease mechanisms and identify innovative therapeutic targets.

## 1. Introduction

The 20th century was marked by a progressive increase in human longevity, attributable mostly to the decrease in deaths associated with infectious diseases. Despite this, a modest increment in lethality resulting from slow damage accumulation disorders, namely cardiovascular and neoplastic, has been registered [1]. Among the neoplastic disorders, mature B-cell lymphoid malignancies are a prominent entity, not infrequently associated with the lack, or overtime loss, of immunological fitness. Mature B-cell non-Hodgkin lymphomas (NHLs) account for 90% of NHL malignancies, with an incidence that has been increasing gradually over time until a recently achieved apparent plateau. Indeed, the incidence of NHL in the United States of America increased from 11.0 to 19.1 per 100,000 persons per year between 1975 and 2019. The incidence of NHL varies according to the histological subtype, race, and ethnicity, with higher rates of disease burden in Caucasians and lower rates in Asian and American natives [2]. Chronic lymphocytic leukemia (CLL) is a chronic lymphoproliferative disorder and the most common leukemia in Western countries, representing up to 30% of all leukemias. The incidence of CLL/small lymphocytic lymphoma has also increased over time, up to 4.9 per 100,000 persons per year in 2021 in the United States [3].

These findings raise awareness about the hypothetical environmental modulation of disease. In fact, these lymphoid malignant entities share a still unexplored epidemiological association with systemic inflammatory disorders [4,5]. During aging, exposures to pathogen-associated molecular patterns, nutrients or their metabolites, and danger-associated molecular patterns resulting from the senescence-related misfolding of proteins and exposure to self-derived nucleic acids promote a pro-inflammatory state. This age-related chronic low-grade inflammation (inflammaging) is characterized by increased levels of pro-inflammatory cytokines, such as interleukin-6 (IL-6) and tumor necrosis factor alpha (TNF-α), and decreased levels of anti-inflammatory cytokines, such as interleukin-10 (IL-10). While the exact mechanisms behind inflammaging are still unclear, it is thought to be caused by a combination of genetic, environmental, and lifestyle factors, including oxidative stress and changes in the gut microbiome. The frequency, intensity, and directionality of the immune response engaged may lead to tissue damage and dysfunction, ultimately leading to cardiometabolic, neoplastic, and neurodegenerative diseases [6]. 

Oxidative stress may promote inflammation directly, by inducing the release of pro-inflammatory cytokines, or indirectly, by damaging cellular components such as DNA, proteins, and lipids, whose components then trigger macrophages and neutrophils [4,5]. Studies have shown that dysregulated redox metabolism plays a role in the development and progression of B-cell malignancies. Reactive oxygen species in the tumor microenvironment can impact not only malignant cells but also bystander cells, including immune cells, influencing therapeutic response and prognosis [5,6].

Herein, we perform a narrative review on the impact of inflammation and oxidative stress on immune cell maturation, particularly those whose dysfunction will give rise to mature B-cell lymphomas. 

### 1.1. Overview of B-Cell Maturation

B cells are key players in the humoral response to pathogens and neoantigens. After migration from bone marrow, precursor cells that have successfully undergone V(D)J recombination and express functional B-cell receptors migrate towards germinal centers, located in secondary lymphoid organs. Germinal centers are complex histological structures with polarity, subdivided in different locations according to the transcriptional programs and putative roles in B-cell maturation: the dark zone, where B cells undergo somatic hypermutation that modulates the variable immunoglobulin’s region; and a light zone, where cell selection based on antigen affinity and class switch recombination occur [7]. The complex maturation of B cells still depends upon the autocrine and paracrine action of different immune players, whose dysfunction may hamper the humoral response generation. Indeed, after migration to the germinal centers, B cells present antigen fragments conjugated with type II major histocompatibility complex molecules to CD4^+^ T cells. These cells will ultimately produce costimulatory and pro-survival signals, as well as engage in displaying chemotactic clues that promote the migration of B cells to the dark zone of germinal centers [8]. In this stage, a transcriptional program dependent upon c-Myc upregulation signaling leads to the expansion of the cells with higher antigen affinity. Then, cells move into the light zone of germinal centers, in which the finetuned selection of centrocytes takes place. This process is essential to select cells that react with exogenous antigens but do not cross-react with self-antigens, minimizing misrecognition and autoimmune phenomena [7]. The light zone compartmentalization is highly dependent on the action of follicular dendritic cells (responsible for the secretion of the B-cell activating factor) and on the cross-regulation between T follicular helper (responsible for pro-survival signals) and T follicular regulatory cells (which antagonize the germinal center reaction expansion by selecting antigen-specific B cells) [8].

### 1.2. B-Cell Malignancies: Nature- or Nurture-Induced Disruption?

Even though genetic or cytogenetic aberrations occur in mature B-cell lymphomas, they are not enough for the arousal of these malignancies. Indeed, populational studies have shown that even healthy subjects carry peripheral blood lymphocytes displaying t(14,18), with absolute counts of this cellular population being tendentiously higher in older individuals. Given that not all these patients develop lymphoproliferative disorders, BCL2 deregulation promoted by t(14,18) is not sufficient to transform lymphocytes [9]. Therefore, it may be hypothesized that the neoplastic transformation of mature B cells will rely not only on germinative and/or somatic mutations or chromosomal translocations, but importantly, on the interaction with the microenvironment and external stimuli. Besides arousing in the germinal center, follicular lymphoma ontogenesis starts in bone marrow, where V(D)J rearrangement failure leads to t(14;18) (q32;q21) in pre-B cells. Such translocation places the BCL2 oncogene under the transcriptional regulation of promoter regions of the heavy chain immunoglobulin (IGH) gene, located in chromosome 14. This results in the overexpression of BCL2, rendering apoptosis resistance in B cells [10]. The assembly of noncontiguous V, D, and J gene segments leads to the formation of a V(D)J exon encoding the variable region of the B cell receptors. This process is mainly mediated by recombination activating gene (Rag) 1 and 2, responsible for the targeting of recombination signal sequences (RSS) flanking each gene segment. Beyond promoting the desirable variety in B-cell receptors, RSS also predisposes to genomic instability that may lead to oncogenesis [11]. After antigen engagement, B cells harboring the translocation are pruned to immunoglobulin gene diversification, B-cell receptor mutation, and subsequent stringent selection depending upon the affinity of the produced antibodies. The increased expression of antiapoptotic BCL2 inhibits the apoptosis of low- to moderate-affinity B cells during the maturation process, promoting the burden of a neoplastic clone that initiates lymphomagenesis [12]. Some authors demonstrated that the majority of the t(14;18) circulating cells (known as follicular lymphoma-like cells—FLLCs) display the phenotype of memory B cells [13], which, contrarily to their physiologic counterparts, are able to re-enter the germinal center reaction upon antigen engagement. These cells experience successive cycles of somatic hypermutation and clonal selection up to the development of cancer precursor cells and overt neoplastic development [14]. This mechanism emphasizes the importance of the frequency rather than the intensity of antigen exposure and stimulation. Therefore, during disease relapse or transformation, only a small proportion of genetic mutations is shared with those present at disease onset. This genetic variation contributes to the divergent evolution and the neoplastic and clinical course heterogeneity between individuals [15]. 

**Figure 1:**
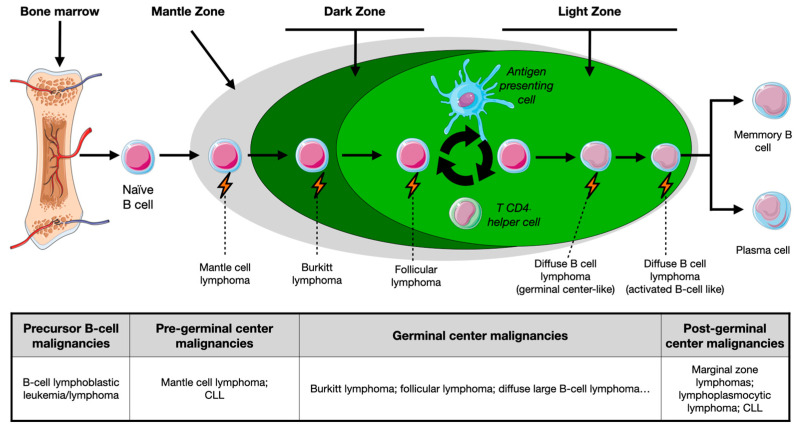
Overview of different stages of B-cell maturation and lymphoproliferative malignancies. Mature B-cell malignancies typically arouse because of cell-intrinsic and cell-extrinsic anomalies by taking advantage of the germinal center biology, notwithstanding that in some instances, naïve B cells carry genetic or chromosomal alterations that arouse earlier than their arrival at germinal centers. CLL—chronic lymphocytic leukemia. Parts of the figure were drawn using pictures from Servier Medical Art (Creative Commons Attribution 3.0).

As an indolent malignancy with a slow progression over time, the persistent redox disruption, pro-inflammatory milieu, and genomic instability over time may render the transformation of follicular lymphoma into a more aggressive disorder, such as diffuse large B-cell lymphoma. An overview of the different stages of B-cell maturation and correspondent lymphoproliferative disorders associated with each stage is illustrated in Figure 1.

## 2. Oxidative Stress and Inflammation in Lymphomagenesis

The chronic pro-inflammatory state, its surrogate biomarkers, and the putative implications in therapeutic management will be reviewed in this section. 

### 2.1. Reactive Oxygen Species

Oxidative stress, induced by environmental exposure, diet, or aging-related senescence, potentiates the development of cardiovascular, degenerative, and neoplastic diseases. The reactive oxygen species (ROSs) act as cell signaling modulators, mainly constituted by oxygen ions including superoxide (O_2_^•−^) and hydroxyl (HO^•^), as well as their reaction products such as hydrogen peroxide (H_2_O_2_) or peroxynitrite (ONOO^−^), with the former being highly dependent on the presence of nitric oxide (NO^•^). Additionally, ROS generation and catabolism play a major role in the innate immune system response. When the balance between pro-oxidant and antioxidant signaling becomes disrupted, there is a propensity for physiological dysfunction and disease development [16].

**Figure 2:**
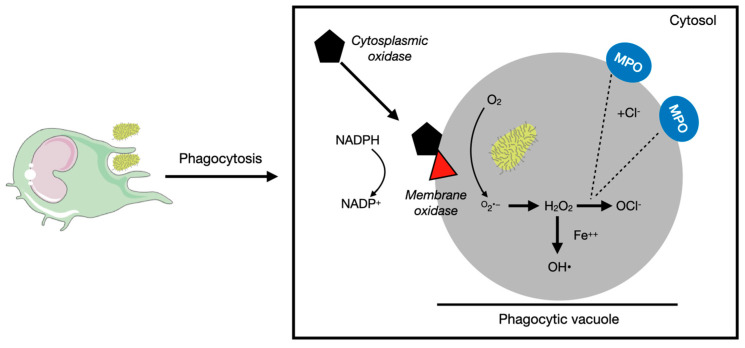
O_2_ reduction generates O_2_^•−^, which by spontaneous dismutation leads to H_2_O_2_. In phagolysosomes, H_2_O_2_ is converted enzymatically by the MPO enzyme (upon fusion of azurophil granules carrying the enzyme with the phagolysosome) to OCl-, responsible for the bactericidal action. In the presence of ferric cation, H_2_O_2_ may also be decomposed, generating HO^•^ (Fenton reaction). Parts of the figure were drawn using pictures from Servier Medical Art (Creative Commons Attribution 3.0).

Considering the physiological role of ROS in the innate immune system, they are master players in the bactericidal effect of neutrophils and macrophages, which is highly dependent on oxidases. The phagocytic oxidase is a complex of proteins present either in the membrane or in the cytosol, responsible for the conversion of oxygen into superoxide. The superoxide radical is extremely toxic and reactive, generating H_2_O_2_ by spontaneous dismutation. Afterward, hydrogen peroxide, by the action of the myeloperoxidase enzyme (present in azurophil granules of inflammatory cells that fuse with the phagolysosome), is converted into hypochlorite, which exerts bactericidal action by halogenation or lipid peroxidation [17], as illustrated in Figure 2.

When certain ROSs interact with cell membranes’ phospholipids, lipid peroxidation may occur. This phenomenon acts in a multitude of steps, ranging from initiation, propagation, and termination, leading to continued cellular damage and triggering an inflammatory response. ROSs act with lipids and generate reactive aldehydes, such as malondialdehyde (MDA) or 4-hydroxy-2-nonenal (4-HNE). Although MDA synthesis occurs by arachidonic acid catabolism, their levels typically increase in situations of higher oxidative stress. MDA is able to interact with other lipids (propagation), but also with DNA and proteins, and this crosslinking property explains its mutagenic and toxic profile. 4-HNE arouses by the oxidation of arachidonic acid, exerting chemotactic clues and leading to the overexpression of pro-inflammatory cytokines, while simultaneously forming covalent adducts with proteins and DNA. Overall, the pro-inflammatory balance of lipidic peroxidation will ultimately depend on the presence of inflammatory cytokines (TNF-α, IL-1ß, and IL-6) and the activation of signaling pathways as NF-κB and ROS-p38/JNK(c-Jun NH2-terminal kinase) [18]. Analogously, protein oxidation may interfere with various metabolic processes and disrupt cellular signaling pathways, while DNA oxidation has been linked to an increased risk of strand breaks and accelerated senescence [16].

Environmental exposures are frequently a source of oxidative stress. High-fat diets, for example, are known to promote the development of non-alcoholic fatty liver disease. Animal experiments have shown that this happens by inducing the expression of inducible nitric oxide synthase (iNOS) in the liver of high-fat-diet-fed animals. Besides increasing the production of reactive nitrogen species, this favors an uncoupling phenomenon in the electron respiratory chain, associated with reduced mitochondrial membrane potential and downregulation of cytochrome C oxidase [19]. Other authors have demonstrated that when long-chain fatty acids such as palmitoyl-CoA are metabolized, the upregulation of the acyl-CoA dehydrogenase occurs. The substrate of this enzyme is dehydrogenated to 2-trans-Enoyl-CoA, and the electrons are transferred to flavin adenine dinucleotide that will then be reoxidized by oxygen-generating H_2_O_2_ [20]. 

Moreover, redox homeostasis is implicated in the immunological response’s modulation. Upon stimulation and antigen recognition, T cells whose metabolism relies mostly on glycolysis and fatty acid oxidation have to adapt accordingly to the context and differentiate into different subsets. This metabolic rewiring relies partially on the production of ROSs by NADPH oxidases and mitochondria electron respiratory chain. At physiological levels and in response to the microenvironment and antigen stimulation of T-cell receptors, ROSs can regulate the expression of transcription factors that influence T-cell fate. While crucial in initiating inflammatory responses, it is essential to maintain a delicate metabolic balance in ROS biogenesis, as an imbalance in the redox state can be detrimental and compromise the host’s ability to mount adequate defense responses [21]. In cancer, ROSs shape the microenvironment and modulate the response to immunotherapies. Higher levels of ROSs are responsible for a reduction in tumor-infiltrating lymphocytes (CD8^+^ cytotoxic cells, natural killer cells, and T helper 1 lymphocytes). Moreover, higher levels of ROSs lead to an exhaustion profile of lymphocytes, a differentiation of CD4^+^ T cells towards a T helper 2 subset, and necrosis-mediated depletion in NK cells. Moreover, given their higher resistance to pro-oxidant states, T-regulatory cells subsist in the neoplastic microenvironment, herein increasing the immune tolerance towards the neoplastic tissues [22]. 

In terms of cell fate, ROSs can induce apoptosis mainly through the induction of cytochrome C release from mitochondria to the cytosol. Upon binding to apoptotic protease activating factor-1 (APAF1), an apoptosome that activates caspase-9 is formed, with the subsequent activation of effector caspases of the intrinsic apoptotic pathway [23]. Otherwise, ROSs are also responsible for the activation of p53, which ultimately renders the transcriptional activation of pro-apoptotic genes in the presence of DNA damage [24].

In terms of cellular biology, the finetuning of intracellular ROS levels relies on enzymes that ultimately catalyze the hydrolysis of H_2_O_2_. Catalase, for example, dismutases H_2_O_2_ to H_2_O and O_2_, while myeloperoxidase uses H_2_O_2_ to generate hypochlorous acid, necessary for phagocyte function [25]. Peroxiredoxins, with thioredoxin as an electron donor, catalyze H_2_O_2_ and lead to the oxidation of cysteine residues of signal transducer and activator of transcription 3 (STAT3), ultimately decreasing its transcriptional activity in the presence of higher levels of ROSs. When oxidized, a disulfide-linked STAT3 unphosphorylated dimer is formed. The cleavage of disulfide bounds with the conversion to transcriptionally activated STAT3 may be achieved by thioredoxin 1, which reduces oxidized cellular components. Given the upregulation of STAT3 in several cancers, the upregulation of thioredoxin 1 in its reduced form in neoplastic tissues is undesirable, as it will prompt downstream signaling mediated by STAT3 [26].

Therefore, ROSs are not infrequently the frontline modulators of the immune response and inflammation. However, given their cross-reaction with other subcellular components, the maintenance of an adequate redox balance is of surmounting importance for one’s health status. The search for environmental modulators of oxidative stress may expand the therapeutic arsenal in the fight against slow damage accumulation diseases, as in the case of the mature B-cell malignancies paradox.

#### 2.1.1. Oxidative Stress as a Modulator of Lymphomagenesis

**Figure 3:**
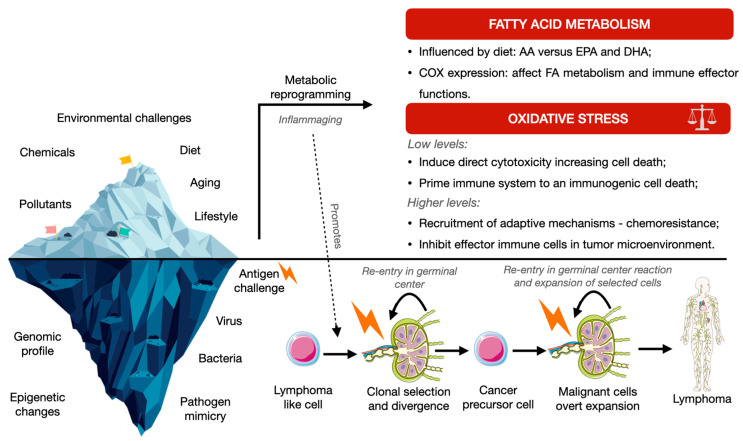
Reactive oxygen species biogenesis and fatty acid metabolism crosstalk in the lymphomagenesis phenomenon. Lymphomagenesis is usually associated with increased levels of ROS, herein exerting inhibitory effects in microenvironment immune cells. Depletion of cancer cells’ antioxidant capacity may become a complementary therapeutical approach. AA—arachidonic acid; DHA—docosahexaenoic acid; EPA—eicosapentaenoic acid; COX—cyclooxygenase; FA—fatty acids. Parts of the figure were drawn using pictures from Servier Medical Art (Creative Commons Attribution 3.0) or PNGAll.com (Creative Commons Attribution 4.0).

The dysregulation of redox homeostasis is implicated in cancer initiation and progression, with impaired neoplastic metabolism and microenvironment reprogramming [27] (Figure 3). 

Cancer cells maintain a delicate balance between O_2_^•−^ and H_2_O_2_ produced by their mitochondria. Researchers have explored the use of cellular antioxidant activity as a potential therapeutic strategy to enhance neoplastic cell death. [28]. Notwithstanding, although some mature B-cell malignancies seem particularly susceptible to pro-oxidant therapeutic approaches, the elevation of ROS levels in the tumor microenvironment may hamper and impair the immunogenic response against neoplastic tissues [29]. Observational studies approaching genetic polymorphisms in genes encoding for oxidative stress showed a consistent and significant tendency toward the disruption of this pathway in hematological malignancies. For example, the nitric oxide synthase polymorphism (NOS2A Ser608Leu), known to confer higher enzymatic activity and iNOS expression, was prevalent among patients with diffuse large B-cell lymphoma or follicular lymphoma. On the other hand, the manganese superoxide dismutase (SOD2 Val16Ala) polymorphism (whose functionality is yet to be described) consistently increased the risk of mature B-cell lymphoma (even across different subtypes) [30]. Epidemiological data showed that AKR1A1 (aldo-keto reductase) polymorphisms in homozygosity were associated with a higher risk of diffuse large B-cell lymphoma development [31]. In another cohort, the presence of GPX1 (glutathione peroxidase 1) and MPO (myeloperoxidase) genetic polymorphisms conveyed an increase in mature B-cell malignancy risk [32]. In a pooled analysis of case–control studies in different populations, homozygosity for the SOD2 16Ala allele was associated with a decreased risk of marginal zone lymphoma, while the GPX1 197Leu allele polymorphism (diminishing glutathione peroxidase activity) displayed a mild association with the mature B-cell malignancy burden (mainly follicular lymphoma) [33]. Despite the general lack of functional studies in patients with lymphoid malignancies, the SOD2 16Ala polymorphism was shown to reduce the levels and the antioxidant activity of superoxide dismutase in a cohort of patients with sickle cell disease [34]. On the other hand, an increase in peroxiredoxins, enzymes with antioxidant activity, in histological samples of untreated follicular lymphoma patients correlated with higher overall survival but not with progression-free survival [35]. 

#### 2.1.2. Oxidative Stress as a Therapeutical Target and Modulator of Treatment Response

Several studies have addressed the role of ROSs in response to therapy. In a cohort of patients with aggressive B-cell non-Hodgkin lymphomas treated with anthracyclines, the MPO rs2243828 polymorphism in homozygosity and NCF4 rs1883112 were associated with increased progression-free survivals (hazard ratio (HR) of 1.87, 95%CI = 1.14–3.06, *p* = 0.013 and 0.66, 95%CI = 0.43–1.02, *p* = 0.06) [36]. This may suggest the immunogenic role of some conventional chemotherapeutic agents and the immune reconstitution priming after treatment, which undoubtedly plays a yet untold story in the biology of disease. Concerning mantle cell lymphoma, in vitro primary cultures and cell lines exposed to bortezomib (a proteasomal inhibitor able to induce proteotoxic stress) displayed an upregulation of endoplasmic reticulum and oxidative stress response pathways. In this experimental setting, the promotion of an antioxidant response in the dependence of Nrf2 (nuclear factor erythroid 2-related factor 2) was associated with chemoresistance, while a lower antioxidant capacity of neoplastic cells was consistent with an increase in NOXA proapoptotic expression [37]. The most instrumental application of redox biology in lymphoma treatment reports to the anthracycline-containing regimens. Doxorubicin is an anthracycline able to induce DNA damage, inhibit type II topoisomerase, and foster an oxidative stress response in the neoplastic cell, ultimately leading to its death [38]. Concerning an experimental model of diffuse large B-cell lymphoma (DLBCL), cytotoxicity was mainly dependent upon anthracyclines’ DNA intercalation in the germinal center-like neoplastic B cells, while neoplastic cells expressing more terminal differentiation (as in activated B-cell like DLBCL), toxicity was more dependent on oxidative stress. In ABC-DLBCL, STAT3 overexpression led to an upregulation of the SOD2 gene, boosting the antioxidant capacity of neoplastic cells and increasing anthracycline resistance [28]. To increase oxidative stress and reduce oxidative antagonization systems in B-cell malignancies, auranofin (AUR) and buthionine-sulfoximine (BSO) were tested in lymphoma cell lines and mantle cell lymphoma primary cultures. AUR acts as a thioredoxin reductase inhibitor (a particularly interesting target given its upregulation in cancer), while BSO is an intracellular glutathione-depleting agent. These two molecules displayed a synergistic effect and induced cytotoxic cell death, at least partially dependent on the inhibition of nuclear factor κB (NF-κB) signaling [39]. In mantle cell lymphoma, the combination of carfilzomib (proteasome inhibitor) and ricolinostat (HDAC6 inhibitor) increased ROS production, promoting JNK activation and cell death. In vitro experiments using a mantle cell lymphoma mice xenograft model exhibited the synergistic action of both drugs in inducing cell death [40]. The metabolic reprogramming of both the tumor microenvironment and neoplastic tissue may render a promising strategy in the near future. The inhibition of the thioredoxin antioxidant system with auranofin (impairing the scavenging of H_2_O_2_) potentiated the anticancer activity of ascorbate both in vitro and in vivo [41,42] when coupled with the exposure or administration of L-ascorbate (autoxidation agent).

Lastly, to corroborate the pre-clinical experiments approaching the oxidative metabolism in mature B-cell malignancies, early trials were developed to determine the safety and efficacy of potential therapeutical targets. A phase II clinical trial using imexon (a pro-oxidant thiol-binding molecule responsible for inducing mitochondrial oxidation with loss of membrane potential and, ultimately, cytochrome C release) was developed in patients with mature B-cell malignancies, achieving an overall response rate of 30%, with responders displaying a higher expression of CD68, GPX1, and SOD2 in pretreatment biopsies [40]. These preliminary findings allow us to hypothesize that oxidative-stress-modifying drugs may be used in combination with conventional treatment regimens in hematological patients.

### 2.2. Fatty Acids

Besides being a source of energy, dietary fatty acids are a structural component of the cell membrane and precursors of signaling molecules that play a key role in a wide spectrum of biological processes. Mice fed with high-fat diets display changes in the gut microbiome and exhibit a higher potential to translocate lipopolysaccharide (LPS) into the bloodstream. LPS is an endogenous toxin present in the bacterial cell wall of Gram-negative microorganisms. When translocated, LPS is able to activate the immune system and initiate an inflammatory response even at very small concentrations. The fat source also influences the absorption of endotoxins, with saturated fatty acids promoting a higher uptake of LPS, probably under lipid-raft-mediated endocytosis. On the other spectrum, ω-3 polyunsaturated fatty acids present in fish oil, such as docosahexaenoic acid (DHA) and eicosapentaenoic acid (EPA), are precursors of anti-inflammatory lipid mediators. DHA induces neutrophil apoptosis and their phagocytosis by macrophages and downregulates TLR4 (Toll-like receptor 4), which functions as an LPS receptor in lipidic rafts [43]. LPS initiates and propagates the inflammatory response through a signaling cascade that involves the activation of the NF-κB pathway, with data prompting the fish-oil-derived fatty acids as inhibitors of this signaling cascade [44]. As is the case for other fatty acids, arachidonic acid (widely present in meat) may be incorporated into the cell membranes. 

There is an accepted correlation between the amount of arachidonic acid accumulation in membrane phospholipids and the ability of inflammatory cells to induce the accumulation of pro-inflammatory eicosanoids such as prostaglandin-E2 (which increases vascular permeability and vasodilation in a cyclooxygenases-dependent way) and leukotriene B4 (produced by 5-lipoxygenase or ALOX5) [43,44]. Not surprisingly, supplementation with fish oil has been shown to increase DHE and EPA, thereby antagonizing the synthesis of pro-inflammatory eicosanoids. Under the action of cyclooxygenases and lipoxygenases, EPA can give rise to five-series leukotrienes that, although pro-inflammatory, have a much lower potency than the four-series generated by arachidonic acid. Both EPA and DHA may be catalyzed by resolvins, negative regulators of the inflammatory response [44].

Ketogenic and caloric restriction diets have been implicated in the reduction of the pro-inflammatory state. A randomized controlled trial was performed to assess the impact of a ketogenic or restriction diet on the peripheral leukocytes’ expression of pro-inflammatory enzymes such as COX-1, COX-2, and ALOX-5 (catalyzers of the synthesis of pro-inflammatory eicosanoids) and ALOX-15 (a catalyzer of the synthesis of anti-inflammatory eicosanoids) in patients with inflammatory-mediated conditions (such as multiple sclerosis) and in healthy controls. Patients on these diets had decreased expression levels of cyclooxygenases and 5-lipoxygenase (ALOX5) as well as a better self-reported quality of life [45]. 

Thereby, it may be concluded that not all fats induce inflammation. However, the circulating levels of some regulatory fatty acids and/or their membrane abundance act as a substrate to, directly or indirectly, influence the inflammatory state. The cross-regulation of dietary fatty acids and the gut microbiome is a matter of active research. Indeed, such crosstalk modulates the translocation of endotoxins, the upregulation of cyclooxygenases and 5-lipoxygenase, and the expression of inflammatory eicosanoids. These processes may challenge the germinal center reaction, imposing a pathological maturation of B cells. Beyond the structural diversity of lipid- and fatty-acid-metabolizing enzymes, lipid rafts and exosomes are also implicated in the pathobiology of lymphoproliferative disorders, although this is beyond the scope of this review. 

#### 2.2.1. Fatty Acids as Environmental Modulators of Lymphomagenesis

The rising incidence of non-Hodgkin lymphomas led to the hypothesis that environmental factors act as triggers in the context of a lack of immunological fitness. Indeed, epidemiological data clearly prompt the consumption of saturated animal fat and obesity as a risk factor for NHL, contrarily to the consumption of vegetables or wholegrains [46]. These observations are of particular interest given the meat-derived arachidonic acid, which, upon metabolization, may shape and modulate immune function [44]. Observational studies have shown that arachidonic acid and its metabolites (pro-inflammatory prostaglandins and leukotrienes) are increased in lymphoma cells and able to promote neoplastic growth and survival while simultaneously promoting angiogenesis and inducing immune suppression [47]. 

COX-2 expression, responsible for the conversion of arachidonic acid into pro-inflammatory eicosanoids and leukotrienes, was shown to be upregulated in patients with Hodgkin lymphoma (70% of cases) and non-Hodgkin lymphoma (up to 57% of cases) [48]. Moreover, tendentiously, the higher the disease stage, the higher the COX-2 expression. Treatment response rates in an observational study ranged from 20.8% in COX-2-positive patients to 70.6% in COX-2-negative patients [48]. In a retrospective observational study, patients exposed to COX-2 inhibitors who underwent an immunochemotherapeutic approach targeting a newly diagnosed DLBCL expressed a higher hazard ratio of survival advantage at one year of follow-up (HR = 0.57, 95% CI 0.43–0.74) [49]. These effects may be related to the immunomodulatory effects of COX-2 inhibition, mainly by reducing the abundance of MDSCs (myeloid-derived stromal stem cells) present in the tumor microenvironment. These cellular components are well known for their immunosuppressive properties, leading to the expansion of T-regulatory cells and the production of ROSs, ultimately impairing the function of effector CD4^+^ and CD8^+^ T cells [50].

Concerning the anti-inflammatory properties of ω-3 polyunsaturated fatty acids, a randomized clinical trial in patients with hematological malignancies addressed the benefits of fish oil supplementation in patients with leukemia or lymphoma and under active treatment. In fact, in those receiving fish oil oral supplementation, the circulating levels of docosahexaenoic acid (DHA) and eicosapentaenoic acid (EPA) were higher, as well as the long-term survival [51]. Beyond these properties, fish-derived polyunsaturated fatty acids were also reported to enhance proapoptotic effects in malignant cells, while concomitantly increasing chemosensitivity [52].

#### 2.2.2. Fatty Acids as a Modulator of Treatment Response and Therapeutical Target

As an oncogenic phenomenon, lymphomagenesis is widely dependent on metabolic reprogramming. This arises from the dependence on fatty acids for organelle biogenesis, signal transduction, and energy generation. For such, an upregulation of the de novo synthesis of fatty acids, in a reaction dependent on NADPH as a reducing agent, is needed. Fatty acid synthase enzyme (FASN) catalyzes the conversion of acetyl-Co-A to malonyl-CoA, which acts as a substrate for palmitate synthesis [53]. FASN was shown to be upregulated in both primary samples and cell lines of DLBCL, with its levels being correlated with the activation of the phosphatidylinositol-3′-kinase (PI3K)/AKT pathway. Indeed, using in vitro approaches, the inhibition of FASN using the C-75 (a synthetic compound) or FASN siRNA was able to promote the dephosphorylation of PI3K and downstream effectors (such as glycogen synthase kinase 1—GSK-1), boosting the intrinsic apoptotic pathway by cytochrome C release to the cytosol [54]. These findings corroborated in primary cultures and cell lines of mantle cell lymphoma prompted the in vitro testing of orlistat (a lipase inhibitor also with irreversible FASN inhibitory properties), able to increase cytotoxicity with a cyclin D1 decrease and a simultaneous increase in the apoptotic rate of malignant cells [55].

### 2.3. Fatty Acids and ROS Cross-Regulation as a Result of Environmental Reprogramming

Fatty acid metabolism and ROSs are interrelated, displaying a cross-regulatory pathophysiology (Figure 3). The interaction between fatty acid metabolism and ROSs happens primarily through the production of the former by β-oxidation. In fact, fatty acid metabolism derivatives, such as acetyl-CoA that enters the tricarboxylic acid cycle, boost the substrate for enzymes such as α-ketoglutarate dehydrogenase, which are ultimately responsible for the genesis of ROSs [56]. On the other hand, other nutritional aspects contribute to the modulation of metabolism and ROS biogenesis, with cellular glutamine depletion in cells, for example, rendering the reduced biogenesis of GSH and lower antioxidant potential, herein increasing cellular levels of ROSs [57]. Concerning lipidic anabolism, long-chain fatty acids are synthesized upon the action of FASN in a series of steps that depend on NADPH. The in vitro inhibition of FASN was shown to increase intracellular ROS [58] in a process that evolves in a positive feedback loop. Indeed, ROS generation leads to the consumption of cytosolic NADPH and to the abrogation of long-chain fatty acid biosynthesis [59]. On the other hand, eicosapentaenoic and docosahexaenoic acids were shown to reduce ROS production (in concordance with its anti-inflammatory profile) and the oxidation of proteins and lipids in plasma, mainly by the upregulation of antioxidant enzymes, such as catalase [60].

## 3. Conclusions

Disease burden relies on the interaction between genomic traits and environmental factors. As a malignant disorder of lymphoid tissue, mature B-cell lymphomas are a result of a stepwise accumulation of cytogenetical and transcriptional abnormalities, not infrequently dependent upon a random antigen-mediated selection in the germinal center, that ultimately leads to the unantagonized growth of a malignant clone. Considering the immune origin of these entities, as well as their dependency on immune players from the tumor microenvironment, the modulation of inflammatory cascades is a promising strategy to understand disease biology, as well as to expand the existing therapeutic armamentarium. 

Presently, anthracyclines and proteasomal inhibitors are drugs exploiting the finetuned equilibrium and tolerance of malignant cells to reactive oxygen species. Notwithstanding, an untargeted approach may be deleterious, given that when ROSs are present at elevated levels, microenvironmental immune cells may become anergic and allow neoplastic growth or even become desensitized to an effective immunotherapeutic approach. On behalf of these principles, recent trials taking advantage of imexon (mitochondrial oxidation inducer) exploited its efficacy in mature B-cell malignancies. These trials have shown promising results in selected patients.

Last but not the least, considering the cross-regulation between fatty acid metabolism and reactive oxygen species biogenesis, it is expected that, in the near future, the repurposing of drugs targeting these pathways, or even the development of new ones, may shape the therapeutic approach to mature B-cell malignancies. 

## Data Availability

Not applicable.
